# IR808@MnO nano-near infrared fluorescent dye’s diagnostic value for malignant pleural effusion

**DOI:** 10.1186/s12931-023-02659-0

**Published:** 2024-01-09

**Authors:** Xiaoqiong Wang, Xingya Yan, Zhipeng Zhang, Chuchu Xu, Fangbin Du, Yanghu Xie, Xiaona Yin, Zubao Lei, Yinling Jiang, Wanchun Yang, Xuan Zhou, Yongsheng Wang

**Affiliations:** 1 Department of Pulmonary and Critical Care MedicineThe Second People’s Hospital of Hefei, Hefei Hospital Affiliated to Anhui Medical University, Hefei, 230011 Anhui Province China; 2 Department of Cardiology, The Second People’s Hospital of Hefei, Hefei Hospital Affiliated to Anhui Medical University, Hefei, 230011 Anhui Province China

**Keywords:** IR808@MnO, Nano-near infrared fluorescent, Malignant pleural effusions, Diagnostic

## Abstract

**Background:**

Malignant pleural effusion is mostly a complication of advanced malignant tumors. However, the cancer markers such as carbohydrate antigen 125 (CA 125), carbohydrate antigen 15-3 (CA 15-3), carbohydrate antigen 19-9 (CA 19-9), and cytokeratin fragment 21-1 (CYFRA 21-1) have low sensitivity and organ specificity for detecting malignant pleural effusion.

**Research question:**

Is IR808@MnO nano-near infrared fluorescent dye worthy for the diagnosis in differentiating benign and malignant pleural effusions.

**Study design and methods:**

This experiment was carried out to design and characterize the materials for in vitro validation of the new dye in malignant tumor cells in the A549 cell line and in patients with adenocarcinoma pleural effusion. The dye was verified to possess tumor- specific targeting capabilities. Subsequently, a prospective hospital-based observational study was conducted, enrolling 106 patients and excluding 28 patients with unknown diagnoses. All patients underwent histopathological analysis of thoracoscopic biopsies, exfoliative cytological analysis of pleural fluid, and analysis involving the new dye. Statistical analyses were performed using Microsoft Excel, GraphPad Prism, and the R language.

**Results:**

The size of IR808@MnO was 136.8 ± 2.9 nm, with peak emission at 808 nm, and it has near-infrared fluorescence properties. Notably, there was a significant difference in fluorescence values between benign and malignant cell lines (p < 0.0001). The malignant cell lines tested comprised CL1-5, A549, MDA-MB-468, U-87MG, MKN-7, and Hela, while benign cell lines were BEAS-2B, HUVEC, HSF, and VE. The most effective duration of action was identified as 30 min at a concentration of 5 μl. This optimal duration of action and concentration were consistent in patients with lung adenocarcinoma accompanied by pleural effusion and 5 μl. Of the 106 patients examined, 28 remained undiagnosed, 39 were diagnosed with malignant pleural effusions, and the remaining 39 with benign pleural effusions. Employing the new IR808@MnO staining method, the sensitivity stood at 74.4%, specificity at 79.5%, a positive predictive value of 69.2%, and a negative predictive value of 82.1%. The area under the ROC curve was recorded as 0.762 (95% CI: 0.652–0.872). The confusion matrix revealed a positive predictive value of 75.7%, a negative predictive value of 75.6%, a false positive rate of 22.5%, and a false negative rate of 26.3%.

**Interpretation:**

The IR808@MnO fluorescent probe represents an efficient, sensitive, and user-friendly diagnostic tool for detecting malignant pleural fluid, underscoring its significant potential for clinical adoption.

## Introduction

Malignant pleural effusion is mostly a complication of advanced malignant tumors [1]. Pleural effusion grows rapidly, and patients often suffer from acute respiratory distress due to the pressure of a large amount of pleural effusion [2]. The prognosis is poor, with a median survival of 3 to 12 months [3, 4]. Malignant pleural effusion is stage IV in cancer staging, and radical resections cannot be performed at this stage. For clinical decision-making, it is therefore crucial to confirm the diagnosis of malignant pleural effusion. In our practice, malignant pleural effusions are definitively diagnosed by exfoliative cell cytology [5]. However, malignant cells and mesothelial cells are morphologically similar to each other, especially when the number of exfoliated cells is low [6]. In addition, the process of cytological analysis is complicated, and the results are highly dependent on the experience of the pathologist, with a strong influence of subjective factors, which makes the results easy to be biased [7]. Cytology sensitivity is only between 20% and 60% [3, 8]. Previous research discovered that cancer markers such as carbohydrate antigen 125 (CA 125), carbohydrate antigen 15-3 (CA 15-3), carbohydrate antigen 19-9 (CA 19-9), and cytokeratin fragment 21-1 (CYFRA 21-1) have low sensitivity and organ specificity for detecting malignant pleural effusion [4, 9, 10]. As a result, there is an urgent need for a safe, quick, and accurate diagnostic approach for distinguishing between benign and malignant pleural effusions.

In recent years, with the superior performance of optical imaging with dyes of near-infrared fluorescence, it has gradually come to the attention of the public [11]. Near-infrared fluorescence has higher tissue penetration than ultraviolet and visible light, and it has non-invasive, good biocompatibility, and tumor-targeting properties, which are used in the fields of tumor optical imaging and tumor photodynamic therapy [12]. Mitochondria are important organelles for maintaining cell survival and play an important role in energy production and apoptosis. However, mitochondria of tumor cells are depolarized and damaged, whereas NIR fluorescent dyes can be specifically adsorbed, so they have tumor-targeting properties but lack the specificity to identify tumor types [11, 13]. Currently, NIR fluorescent dyes include IR806, IR808, IR783, IR820, and so on [14]. By reviewing the literature, it was reported that a team applied IR808 dye in the identification of benign and malignant pleural effusion and concluded that IR808 has advantages in identifying malignant pleural effusion. However, the sample size of this study was only 28 cases, which was only cross-sectional study, and its diagnostic ability was not reflected by receiver operating characteristic (ROC) curve [15]. Moreover, near-infrared fluorescent dyes, such as IR808, generally have the disadvantages of poor photostability, poor water solubility, and easy degradation [16], so we urgently need to design a hydrophilic, low-toxicity, and superior photostability dye probe to improve the ability to identify tumor cells.

Therefore, the present experimental design uses MnO as a nanocarrier in which the IR808 dye is embedded to protect its fluorescent properties while increasing its photostability from degradation, and the inclusion of MnO groups can increase its water solubility [13, 17]. The diameter and fluorescence spectra of IR808@MnO nanoparticles were first measured by material characterization. Secondly, we selected malignant and benign cells to verify in vitro whether IR808@MnO has tumor-targeting properties, as well as to map out the optimal concentration and duration of action. Then, we selected pleural fluid from patients with a definite diagnosis of malignant pleural effusion and used IR808@MnO to map its optimal concentration and time of action. Finally, 106 patients with indications for thoracoscopic operation in our hospital were selected for fluorescent dyes, and the pathological results of thoracoscopy were used as the gold standard to go over the diagnostic ability of the new methods.

## Experimental section

### 1Heptamethine dye and pleural fluid samples

The heptamethine dye IR808 was purchased by Xi’an KaiXin Biotechnology Co., Ltd. Meanwhile, IR808@MnO was technology synthesized by the same company. Before use, both were kept in – 20 °C. All pleural fluid samples were collected from the Department of Respiratory and Critical Care Medicine, the second People’s Hospital of Hefei, Hefei Hospital affiliated with Anhui Medical University. All pleural fluid specimens were obtained for internal thoracoscopy and were all pathologically diagnosed. Pathology report issued by the same Hospital.

### Cell culture and cell lines

All human benign and malignant lines in this trial were sponsored by CAS key laboratory of Nano-Bio Interface, Suzhou Institute of Nano-Tech and Nano-Bionics, Chinese Academy of Sciences and Center for Clinical Mass Spectrometry, college of Pharmaceutical Sciences. Malignant tumor cells were kept viable in growth medium (high-glucose DMEM with 10% FBS and 1% PS), including human lung adenocarcinoma cells (YB-71596HC, CL1-5), human non-small cell lung cancer (ORC0015, A549), human breast cancer (SNL-061, MDA-MB-468), human glioblastoma (SNL-096, U-87MG), human kidney cancer (SNL-369, MKN-7), human cervical cancer (ORC0086, Hela). Normal human tissue cells were cultured in growth medium (RPMI 1640 with 10% FBS and 1% PS), including human epithelial cells (C6106, BEAS-2B), human umbilical vein endothelial cells (CL-0122, HUVEC), human fibroblasts (CP-H183, HSF), human vascular endothelial cells (CL-0310,VE).

### Synthesis of IR808@MnO

We based our synthesis on the N-terminal (A1A2A3) and the C-terminal (An). Starting with An at the C-terminus Weigh the resin Fmoc-An-Resin according to the required amount and soak it in the reaction column for 30 min. After the resin had been soaked for 30 min, the solution was drawn off and then deprotected, and the deprotection reaction was carried out with piperidine for 30 min. Within 30 min of deprotection, the number of amino acids, condensate, and NMM needed for the reaction at each step was calculated based on the amounts made, and then the weighing of the next amino acid step, Fmoc-An-1, was performed. After 30 min of deprotection, the piperidine was drawn off and then washed six times with DMF. The color of the deprotection was tested after six washes and recorded. After washing, deprotection, and testing, the peptides were added in the order in which they were weighed, then a small amount of reaction solution was added, followed by alkali and NMM. The reaction time was recorded as 30 min with DCM. After thirty minutes of reaction, the reaction solution was drawn off, washed three times with DMF, and then tested to check if the reaction was complete. Once the test had been connected, i.e., steps 2–6, it was repeated to connect the next amino acid. If the test was not passed, soak it up with DCM and then continue connecting the amino acids in this step without removing the protection until they are connected. The final synthetic IR808@MnO concentration was 32 mg/ml.

### In vitro uptake and accumulation of IR808@MnO in normal and malignant cells

To clarify the optimal dose and duration of action. We chose adenocarcinoma cells, the most common type of malignant pleural effusion. Human lung adenocarcinoma cells (YB-71596HC, CL1-5) were cultured at a density of 2 × 10^6^ in a six-hole dorsal plate, and incubated at 37 °C with 5% CO2 for 24 h. All cells firmly adhered to the wall proliferate. 2 ml of culture fluid were also present in each well plate. We divided the chemically synthesized IR808@MnO into five concentration gradients of 1 μl, 2 μl, 5 μl, 10 μl, and 20 μl. The concentration values at which the best color development was observed were used as a reference. Meanwhile, another group of CL1–5 was cultured for different times (5 min, 10 min, 30 min, 1 h, 12 h). Five concentration gradients of dyes were mixed with the cell cultures and placed in the incubator for 30 min of coincubation. They were fixed with 4% paraformaldehyde for 5 min after two washes of PBS. After that, images were recorded by a NIR fluorescence small animal live imager (IVIS Lumina III, LAB 15006).

We selected 5 μl and 30 min as the ideal concentration and timing of action based on the fluorescence results. In a six-hole dorsal plate, all cells were cultivated at a density of 2 × 10^6^, and they were all left to incubate for 24 h at 37 °C with 5% CO_2_.All cells that are firmly attached to the wall multiply. There was also 2 ml of culture fluid in each well of the plate. The chemically created IR808@MnO was separated into 5 μl and 30 min. After two PBS washes, they were fixed with 4% paraformaldehyde for 5 min. A NIR fluorescence small animal live imager (IVIS Lumina III, LAB 15006) was then used to capture images.

### Assessment of IR808 and IR808@MnO in malignant pleural effusion

**Fig. 1:**
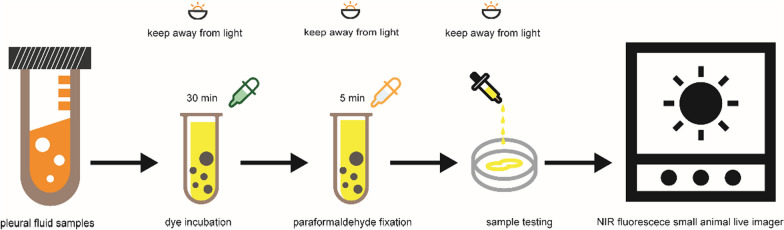
Diagram of the experimental process

Pleural fluid was obtained from 50 ml of pleural fluid collected on the same day, transported to the laboratory in a cold chain at 4 °C, and given immediately by centrifugation at 1200 rpm for 15 min. The upper layer of pleural fluid was discarded, and the lower layer of cells was retained. During the experiment, we found that the cells of malignant pleural fluid origin adhered severely, which affected the results of the experiment, so urokinase-type plasminogen activator (uPA) (10,000 u) was added to the pleural fluid to release the pleural fluid adhesions [18]. The cells were reconstituted in 2 ml of PBS and distributed in six-well plates. The cells were co-incubated with formaldehyde for 30 min. Washed twice in PBS, fixed in paraformaldehyde for 5 min, washed twice in PBS, stored at 4 °C, and kept away from light. The IR808@MnO and IR808, another typical NIR heptacene towards malignancy, were incubated with pleural fluid samples. The procedures followed the pattern presented above. Figure 1 summarizes the entire diagnostic procedure.

### Statistical analyses and histological

All pleural fluid was collected from patients undergoing thoracoscopy at The second People’s Hospital of Hefei, Hefei Hospital Affiliated to Anhui Medical University. All collected pleural fluid had pleural fluid exfoliation cytology pathology as well as thoracoscopic pathology. Of these, the diagnosis of thoracoscopic pathology was the gold standard for diagnosis. All patients signed an informed consent form agreeing to the use of pleural fluid specimens for testing. The ethics committee of our hospital approved the experiment. The ethics number is V2.0/20210112.

**Fig. 2:**
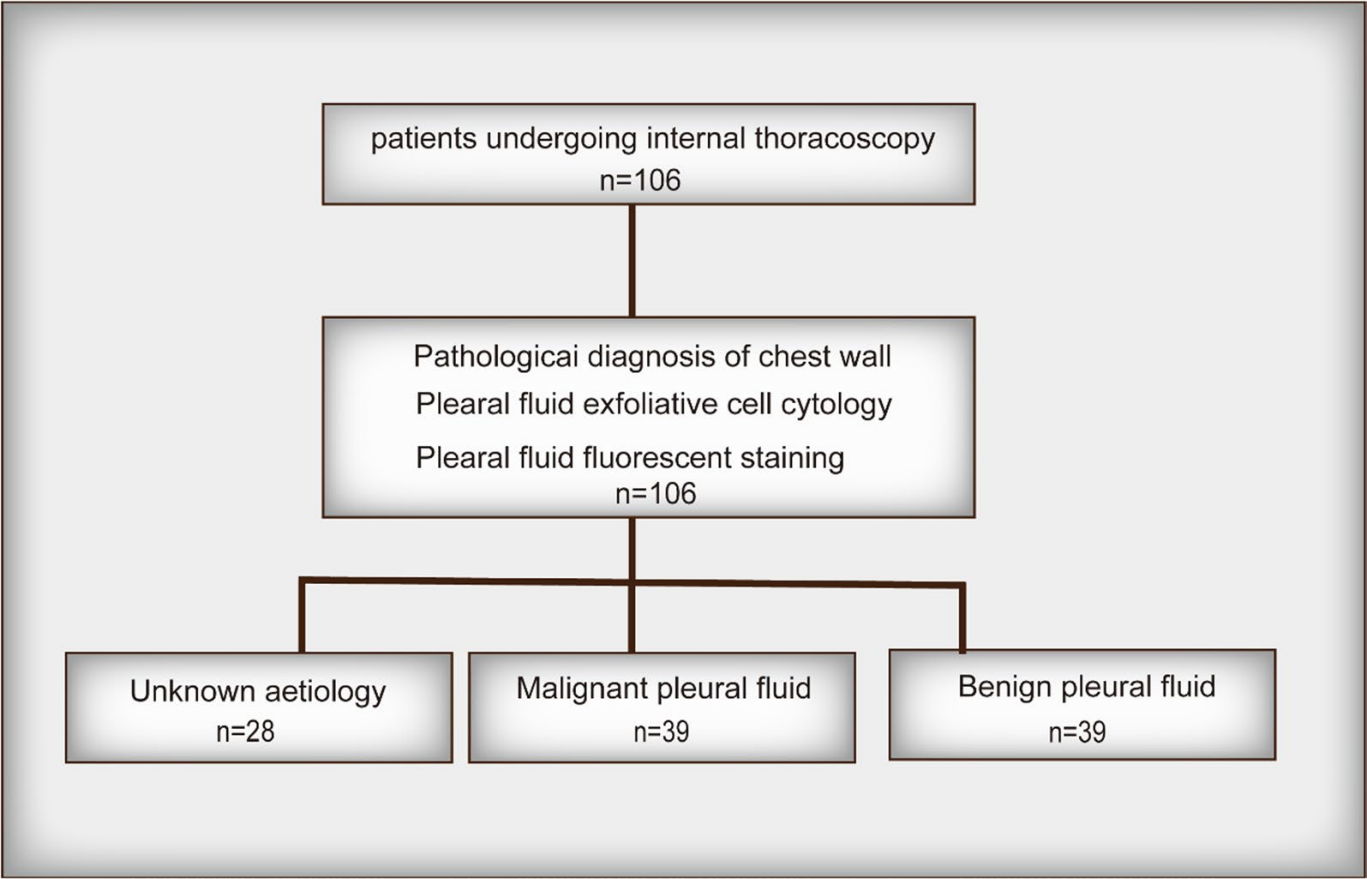
Pleural fluid samples selection process

We selected 116 patients with indications for thoracoscopy for enrollment, excluding those with contraindications to thoracoscopy, including severe hypoxaemia, arrhythmias, abnormal coagulation mechanisms, recent myocardial infarction, unstable angina, aortic coarctation, renal failure, bronchial asthma, tracheotomy, tracheal intubation, severe respiratory failure, mental retardation, and physical weakness unable to cooperate with patients. All patients were stained for pleural fluid fluorescence, pleural fluid exfoliation cell cytology, and pleural pathological diagnosis of chest wall. Of these, patients were classified by pathological diagnosis of chest wall into 28 with unknown aetiology, 39 with malignant pleural fluid, and 39 with benign pleural fluid. All the pleural fluid was stained for live cells (Fig. 2).

We collected all experimental data using Microsoft Excel, including baseline information of enrolled patients and the fluorescence intensity and fluorescence absorbance of IR808@MnO, as well as results of pleural effusion exfoliative cytology examination. Data processing was performed using R version 4.3.1. Data conforming to normal distribution are represented by mean ± standard deviation, and intergroup comparisons are made using t-tests; For non-normally distributed quantitative data, the median P50 (P25, P75) was used for representation, and the Mann–Whitney U test was employed for inter-group comparisons; categorical variables were expressed as percentages, with inter-group comparisons using the chi-square test. Graphical reproduction of the data was completed using GraphPad Prism 9 and R version 4.3.1. In all statistical tests, a two-sided p-value of less than 0.05 was considered statistically significant.

## Results and discussion

### Characterization and synthesis of IR808@MnO

**Fig. 3:**
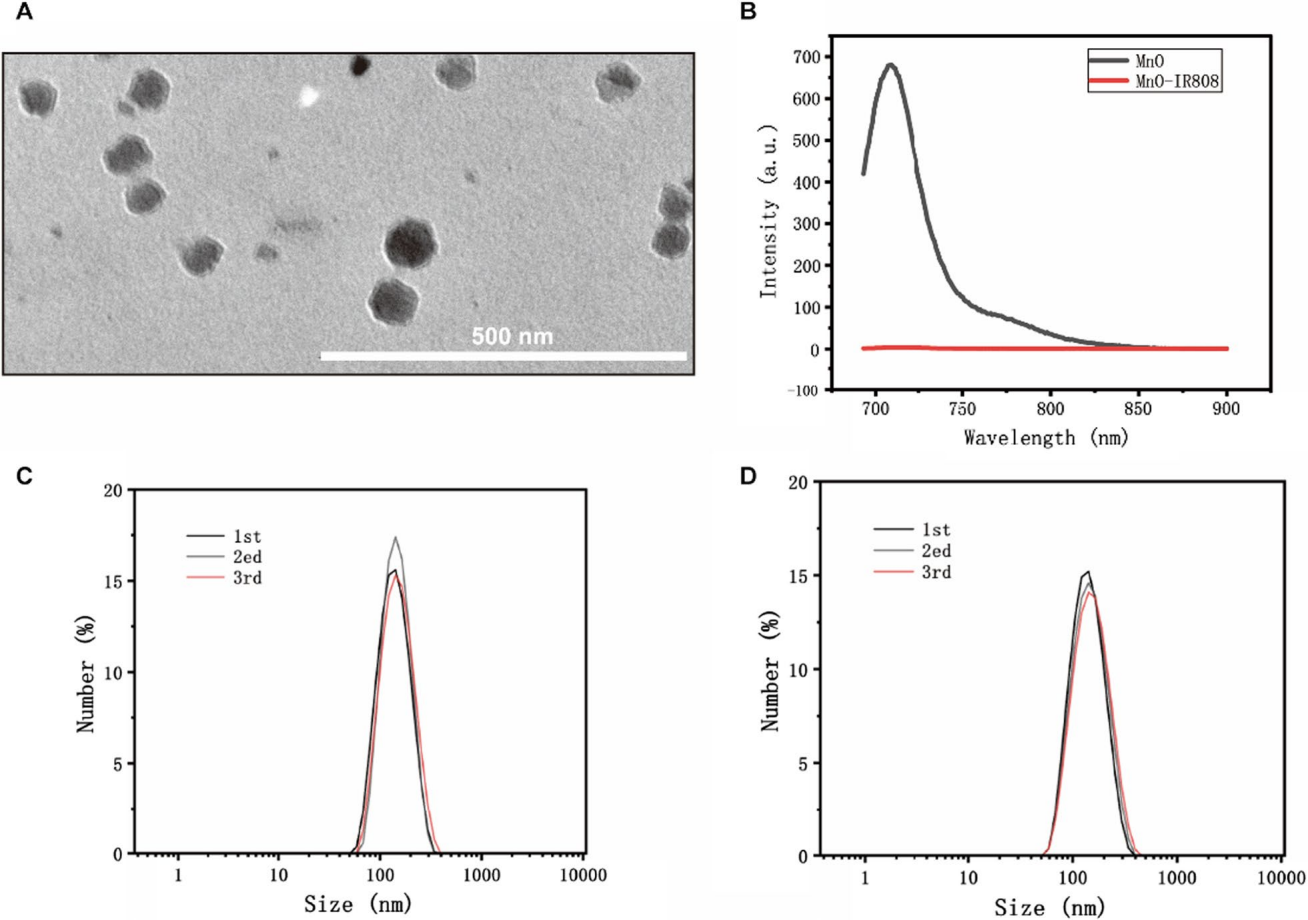
**A** Electron micrographs of IR808@MnO with an average particle diameter of 136.8 ± 2.9 nm. **B** Fluorescence spectrogram of IR808@MnO, MnO does not have fluorescence emission property and the emission peak of IR808@MnO is at 808 nm. **C** The hydrated particle size of MnO is 130.5 ± 3.4 nm. **D** The hydrated particle size of IR808@MnO is 136.8 ± 2.9 nm

Although IR808 is water-soluble, it has poor stability and is readily agglomerated at room temperature. Its caking characteristics remain unchanged when dissolved in DMSO solvent. The precision of experimental data can be impacted by repeated freezing and thawing. This unfavorable scenario can be resolved using doped biocompatible coatings. Divalent metal ions (M = Mn, Co, Ni, and so on) have the potential to produce friendly compounds [19]. To obtain IR808@MnO fluorescent dye, magnetic manganese oxide and IR808 were designed as the core dyes. As shown in Fig. 3b, the synthesized IR808@MnO dissolved in DMSO was stable and did not form agglomerates.

The 200 kV projection electron microscopy showed that the size of IR808@MnO particles matched the size of the dynamic light scanning test particle size, 136.8 ± 2.9 nm. Meanwhile, the dynamic particle size scanning showed that modification of IR808 does not significantly change the size of the nanoparticles and that the hydration of the MnO particles (130.5 ± 3.4 nm) and the MnO-IR808 (136.8 ± 2.9 nm) particle sizes were basically the same. The fluorescence spectra showed that MnO did not have the fluorescence emission property, and the emission peak of IR808 was at 808 nm. The fluorescence spectra of the modification product, IR808@MnO, also showed that the emission peak was at 808 nm, so the modification of IR808 on the surface of manganese oxide particles would not interfere with the fluorescence emission property of IR808. Elemental analysis shows that there is an obvious distribution of manganese and oxygen elements in IR808@MnO particles and relatively few carbon and nitrogen elements, which meets the requirements of manganese oxide-modified particles.

### IR808@MnO: near-infrared average radiant efficiency in benign and malignant cells

**Fig. 4:**
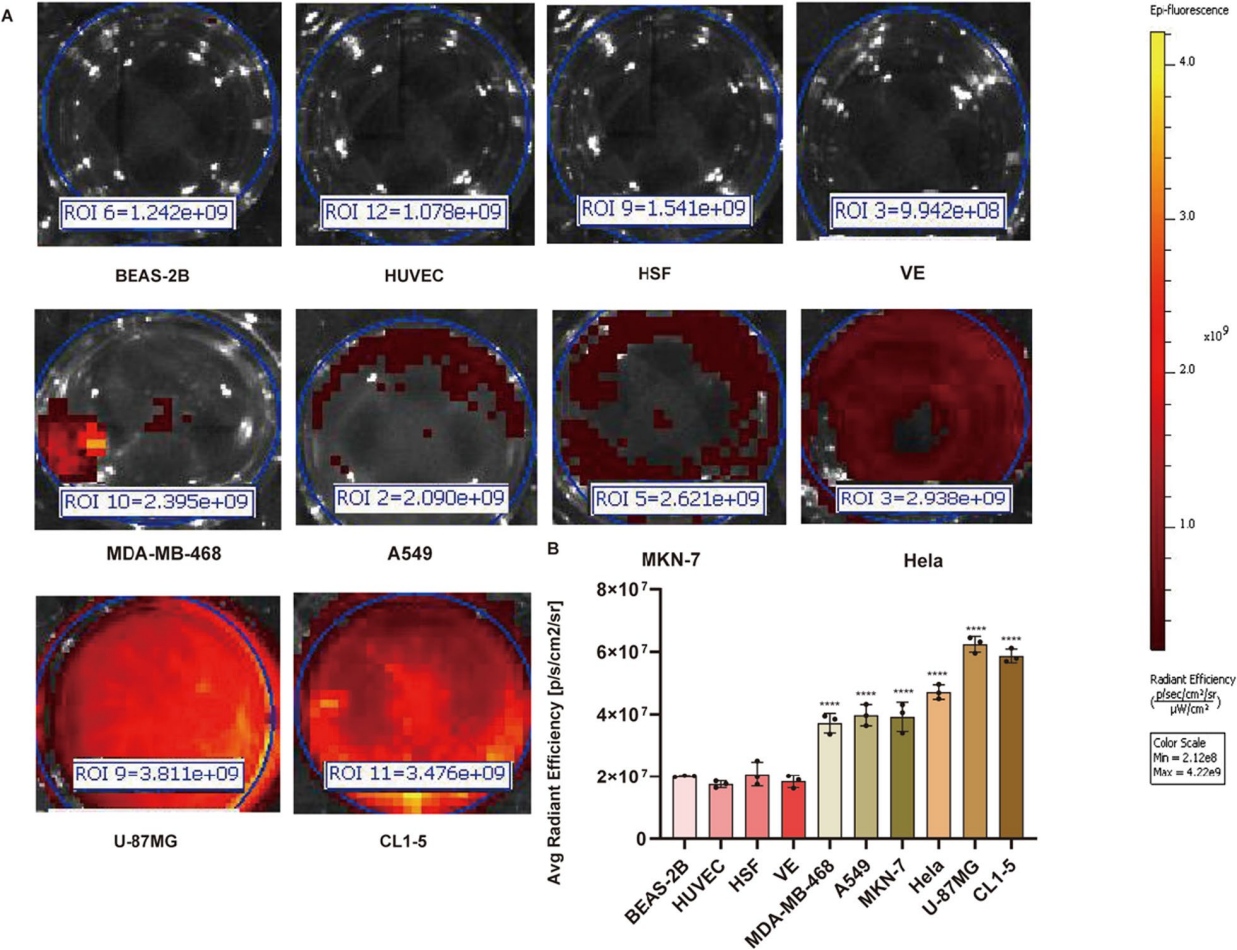
**A** Normal human cells (BEAS-2B, HUVEC, HSF, and VE) showed no uptake of IR808@MnO after incubation. Human malignant cell lines (CL1-5, A549, MDA-MB-468, U-87MG, MKN-7, and Hela) showed significant uptake of IR808@MnO under similar staining and imaging conditions. Results are shown with images obtained from IR808@MnO staining (NIR). All images were acquired by an NIR fluorescence small animal live imager. **B** It showed the average radiant efficiency values of different tumor cells. There was a significant difference between the values of malignant tumor cells and benign cells (p < 0.0001), the average radiant efficiency of different malignant tumor cells were different, and the values of U-87MG and CL-5 were significantly different from those of the other four groups of tumor cells (p < 0.0001)

Our pre-experiments identified the optimal cell concentration and incubation time of 2 × 10^6^ per hole and 30 min, respectively, and the average radiant efficiency per was measured by an NIR fluorescence small animal live imager as shown in Fig. 4A. The fluorescent filter sets (excitation, 740–845 nm; emission, 740–830 nm). Malignant cells included CL1-5, A549, MDA-MB-468, U-87MG, MKN-7, and Hela. Benign cells included BEAS-2B, HUVEC, HSF, and VE. The average radiant efficiency values of all malignant cells were higher than those of benign cells, and the difference was statistically significant (p < 0.0001). However, there was no statistical difference between the values of benign cells BEAS-2B, HUVEC, HSF, and VE. Within the malignant cell group, there was a significant difference between Hela and U-87MG values (p < 0.0001), a significant difference between Hela and CL1-5 (p < 0.01), a significant difference between MEN-7 and CL1-5 and U-87MG values (p < 0.0001), and a significant difference between CL1-5 and A549 and MDA-MB-468 values (p < 0.0001), A549 and U-87MG values (p < 0.0001), and MDA-MB-468 and U-87MG values (p < 0.0001).

As shown in the Fig. 4 values, IR808@MnO aggregates in malignant tumor cells and has tumor-targeting properties. The reason why a certain value of fluorescence existed in the benign cell group was that we repeatedly washed with PBS five times before and after the staining process and fixed in paraformaldehyde once, but there was still a certain amount of fluorescence adsorbed on the surface of the plate, but its value was very low and not visible to the naked eye. The values were significantly higher in the malignant cell group, indicating that the fluorescent dyes were concentrated in malignant cells for color development, especially in human lung adenocarcinoma cells, human cervical cancer, and human glioblastoma. It had been demonstrated that the fluorescent dye IR808 exhibited tumor targeting capabilities, allowing for the observation of tumors in near-infrared fluorescence [12]. Herein, a dye‐anchored manganese oxide nanoparticle (IR808@MnO) could effectively target the mitochondria of tumor cells [13], and the MnO nanoparticle IR808 was encased inside of it, shielding the fluorescent dye from deterioration and enhancing light stability [14].

The average radiant efficiency fluorescence values for CL1-5 and U-87MG were (5.87 ± 0.22) × 10^7^ and (6.24 ± 0.25) × 10^7^, respectively. The average radiant efficiency fluorescence values for MDA-MB-468 and A549, MKN-7, and Hela were (3.72 ± 0.31) × 10^7^ and (3.98 ± 0.34) × 10^7^, (3.92 ± 0.47) × 10^7^ and (4.71 ± 0.23) × 10^7^. The near-infrared fluorescent dye IR808 was a lipophilic cation that was preferentially enriched in the mitochondria of tumor cells, which had a higher membrane potential than normal cells, to achieve a significant signal for in vivo imaging due to its resistance to autofluorescence and its deeper penetration [20]. And because this transport ventricular process was energy-intensive, it could only be achieved by living cells and could also distinguish dead cells from apoptotic cells based on fluorescence [21]. Because we knew that the PH values of different tissue sites in an organism fluctuate greatly and the PH values of different tumor cells vary greatly [22], the fluorescence produced by the same fluorescent dye was determined by the PH responsiveness of different tumors, which explained why the difference in fluorescence values of IR808@MnO between different tumor cells was statistically significant.

### Impact of IR808@MnO on concentration and time tests

**Fig. 5:**
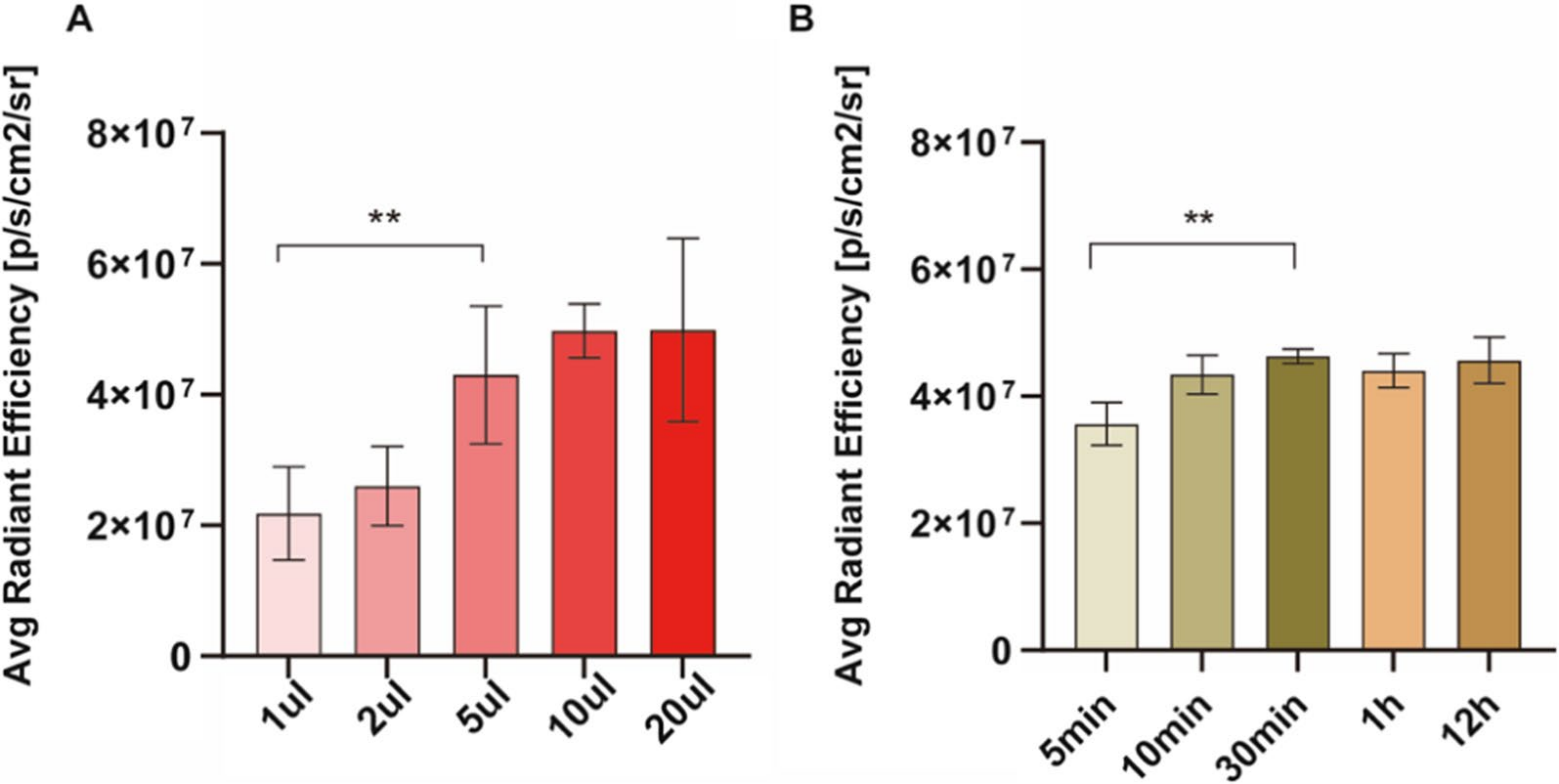
To further optimize the dose and time, 2 × 106 A549 cells were chosen to incubate with IR-808@MnO at the final concentrations of 1 μl, 2 μl, 5 μl, 10 μl, and 20 μl for 30 min (**A**), and time points of 5 min, 10 min, 30 min, 1 h, and 12 h at 5 μl (**B**). The images obtained by staining with IR-808@MnO are shown. The average radiant efficiency values under different conditions were statistically analyzed. All images were acquired by an NIR fluorescence small animal live imager

Pleural metastases from adenocarcinoma of the lung account for the majority of causes of malignant pleural effusions. For the human lung adenocarcinoma cell line, we mostly chose A549 as the cell line model for establishing malignant pleural effusion [23–25]. In order to determine the optimum and minimum IR808@MnO concentrations showing the best fluorescence effect, five cell concentration gradients of 1 μl, 2 μl, 5 μl, 10 μl, and 20 μl were chosen. We found that the mean fluorescence intensity at 5 μl was (3.86 ± 0.90) × 10^7^, which was significantly different from 1 and 2 μl (p < 0.01), and the difference was not statistically significant as the concentration increased, so the best and lowest dye concentration for IR808@MnO is 5 μl (Fig. 5A), which gives the best fluorescence and saves costs.

For the optimal action time, we chose a 5 μl concentration of IR-808@MnO and A549 co-incubated for 5 min, 10 min, 30 min, 1 h, and 12 h. We found the difference between 5 and 10 min to be statistically significant. With the extension of time, even 12 h, the fluorescence color development effect did not change significantly. Therefore, the same 5 μl concentration and 30 min incubation were chosen for the subsequent validation of the clinical samples. In clinical practice, we needed a 24-h report to determine the benignity or malignancy of pleural fluid by exfoliative cell cytology. While we used NIR fluorescent dye, the incubation time was only 30 min, plus the fixation and detection time was less than 1 h, which greatly reduced the time of report issuance.

### Concentration and time trends of IR808@MnO in malignant pleural effusions

**Fig. 6:**
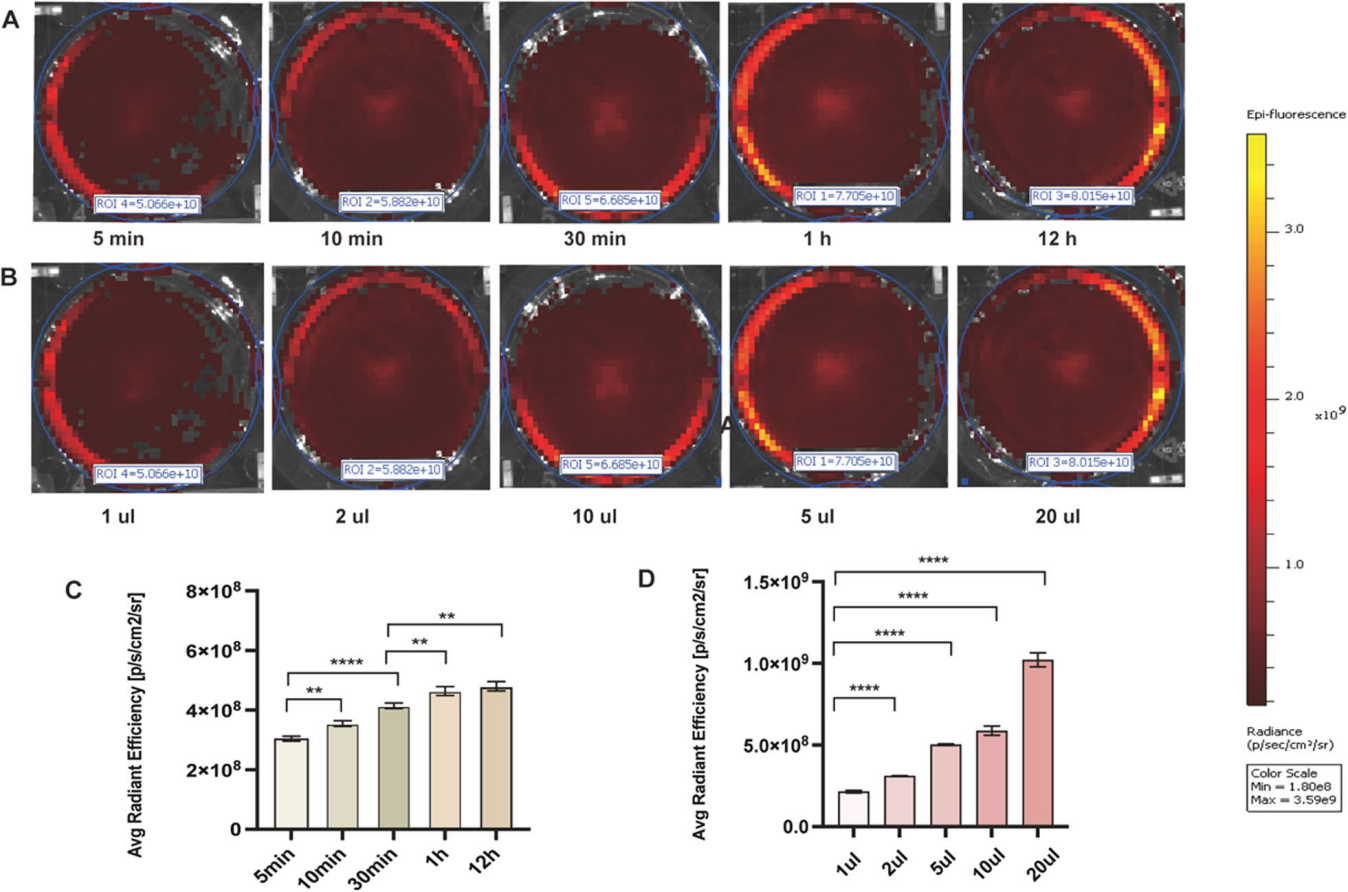
**A** and **B** The average radiant efficiency of malignant pleuroperitoneal effusion (hydrothorax and ascites) with IR808@MnO at different time points (5 min, 10 min, 30 min, 1 h, 12 h) and at different concentrations (1 μl, 2 μl, 5 μl, 10 μl, 20 μl). **C** It showed the average radiant efficiency fluorescence value of malignant pleural fluid cells at different times. **D** It showed the average radiant efficiency fluorescence value of malignant pleural fluid cells at different concentrations

Figure 5 shows the in vitro cell experiments, but we do not know whether fluorescence chromatography can also be performed for human malignant pleural effusion or what the optimal concentration and time are. We chose a patient with pleural metastasis of lung adenocarcinoma, and the exfoliated cells in the pleural fluid were similarly adenocarcinomas. 50 ml of malignant pleural fluid was retained and subjected to centrifugation at 1200 rpm for 15 min. and co-incubation with IR808@MnO. In order to determine the IR808@MnO concentration and the lowest concentration for optimal fluorescence, we chose five cell concentration gradients of 1 μl, 2 μl, 5 μl, 10 μl, and 20 μl. We found a significant difference (p < 0.0001) for concentrations above 5 μl, with an average fluorescence value of (5.03 ± 0.49) × 10^8^ at the 5 μl concentration. Therefore, the optimal and minimum dye concentration for IR808@MnO was 5 μl (Fig. 6D), which provided both optimal fluorescence and cost savings. However, we found that the average fluorescence value in human pleural fluid was about 10 times higher than that of the in vitro experiments with the A549 cell line, which may be due to the fact that other cells, such as fibroblasts, erythrocytes, and other admixed cells, were included in the human pleural fluid. This is close to the average fluorescence intensity value of IR808 in malignant pleural effusion in Ying Tian’s article [15].

Also, for optimal time of action, we chose a 5 μl concentration of IR808@MnO to be incubated with the malignant pleural effusion of this patient for 5 min, 10 min, 30 min, 1 h, and 12 h. We found that there was a significant difference in the mean fluorescence intensity when the incubation time was 30 min (p < 0.0001), and the mean fluorescence intensity was enhanced when the incubation time was increased to 1 h and 12 h, but there was no significant difference between 1 and 12 h. Because IR808@MnO stains living cells, a long incubation time can affect cell activity and even cellular cells. Therefore, we chose the same 5 μl concentration and 30 min incubation time for subsequent clinical sample validation.

### Diagnostic ability of the new IR808@MnO staining method for malignant pleural effusions

We enrolled 106 patients with pleural effusion of unknown aetiology eligible for thoracoscopic manipulation who were stained with the new IR808@MnO method, while the pleural fluid was sent to our pathology department for pleural fluid cytology. The gold standard was the pathology report of the wall pleura of the thoracoscopic operation. Its report was interpreted as malignant for malignant pleural effusion and benign for benign pleural effusion, while the interpretation of unknown was defined as pleural effusion that could not be clearly diagnosed. Statistically, 106 enrolled patients had 39 malignant pleural effusions, 39 benign pleural effusions, and 28 unexplained pleural effusions. 26 of the 39 malignant pleural effusions were adenocarcinomas, 5 small cell lung carcinomas, 4 squamous carcinomas, 1 malignant tumor of epithelial origin, 1 lung epithelial tumor origin, and 2 pleural mesotheliomas. Of the 39 benign pleural effusions, 29 were tuberculous pleurisy, 1 was leakage due to heart failure, and 9 were inflammatory pleural effusions and pyothorax.

**Fig. 7:**
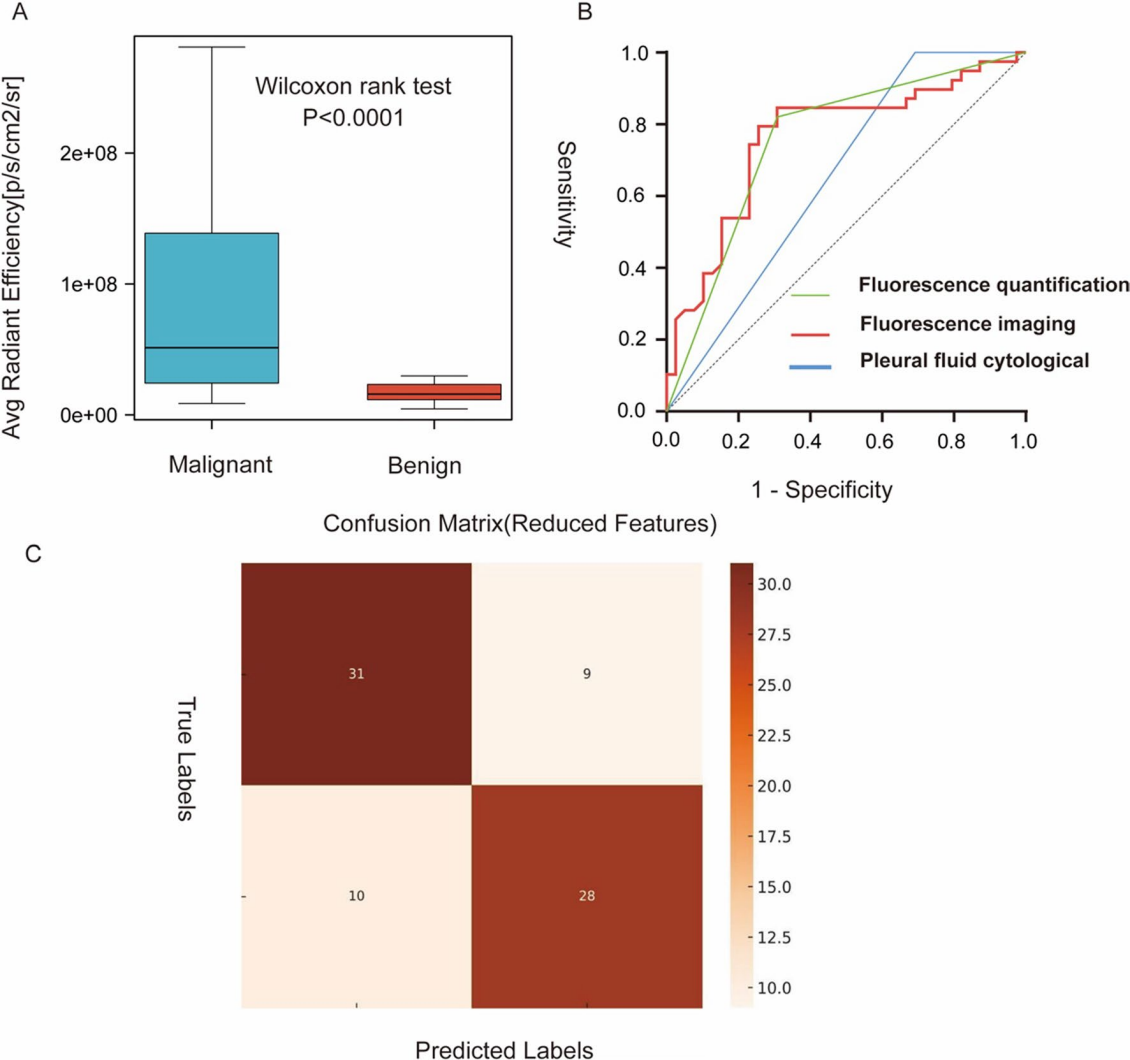
shows a box plot of the average radiant efficiency fluorescence value of malignant pleural effusion and benign pleural effusion with a statistically significant difference. **B** showed a ROC curve of IR808@MnO NIR fluorescence for malignant pleural effusion. **C** showed a confusion matrix for predicting the benignity or malignancy of pleural effusion using the fluorescence value as the indicator

To evaluate the diagnostic ability of the dye for malignant pleural effusions in patients, we conducted baseline data collection for 78 patients. This includes age, gender, and the presence of chronic diseases such as hypertension, diabetes, heart disease, and multiple cerebral infarctions, as well as the results of pleural fluid cytological, fluorescence imaging, and fluorescence quantification (Table 1). There are no statistically significant differences between the two groups in terms of age, gender, and chronic underlying diseases (p > 0.05). However, significant differences are observed in fluorescence imaging, fluorescence quantification, and pleural fluid cytological (p < 0.001). We use R software (version 4.2.1) to compare the fluorescence values between the two groups. Box plot visualization is performed with the ggplot2 package, and with the removal of outlying points, there is a significant difference in the mean fluorescence values between the malignant and benign groups (Fig. 7A). We conduct a qROC analysis on the fluorescence imaging, fluorescence quantification, and pleural fluid cytological using R software; the area under the ROC curve for fluorescence quantification is the highest (Fig. 7B). The area under the ROC curve for fluorescence quantification is 0.762 (95% CI: 0.652–0.872). The area under the ROC curve for fluorescence imaging is 0.762 (95% CI: 0.652–0.872). The area under the ROC curve for pleural fluid cytological is 0.762 (95% CI: 0.652–0.872) (Table 2). In this study, we determined the optimal cut-off point as a fluorescence intensity value of 2.5145 × 10^7^ based on ROC curve analysis, and used it as the classification threshold for distinguishing between benign and malignant samples. The confusion matrix, constructed using this threshold, revealed a positive predictive value (precision) of 75.7%, a negative predictive value of 75.6%, a false positive rate (the proportion of benign samples incorrectly classified as malignant) of 22.5%, and a false negative rate (the proportion of malignant samples incorrectly classified as benign) of 26.3% (Fig. 7C). It indicates that the accuracy of the new method of IR808@MnO fluorescence staining is better.

**Table 1:** Comparison Table of Baseline and Observational Indicators for Dual Patient Groups

Variant	BPE (n = 39)	MPE (n = 39)	χ^2^/Z	P
Age (years)	73 (64, 83)	61 (54, 80)	− 1.530	0.126
Sex			1.338	0.355
Male	26 (66.7)	21 (53.8)		
Female	13 (33.3)	18 (46.2)		
Chronic disease history			2.519	0.173
Yes	15 (38.5)	22 (56.4)		
No	24 (61.5)	17 (43.6)		
Pleural fluid cytological			14.182	< 0.001
Positive	0 (0)	12 (30.8)		
Negative	39 (100)	27 (69.2)		
Fluorescence imaging			20.856	< 0.001
Positive	7 (17.9)	27 (69.2)		
Negative	32 (82.1)	12 (30.8)		
Fluorescence quantification (10^7^)	5.13 (2.30, 1.50)	1.59 (1.14, 2.42)	− 3.978	< 0.001

BPE: Benign Pleural Effusion Group; MPE: Malignant Pleural Effusion Group; Chronic medical history mainly includes chronic diseases such as high blood pressure, diabetes, heart disease, cerebral infarction, etc. Pleural fluid cytological: traditional pleural effusion cytology examination and diagnosis; Fluorescence imaging: the fluorescence absorption of IR808@MnO in malignant cells; Fluorescence quantification: the fluorescence intensity of IR808@MnO

**Table 2:** Analysis of predictive efficacy for different indicators

Indicator	Cutoff	Sensitivity (%)	Specificity (%)	J	AUC	95%Cl	P
Fluorescence quantification	25,145,000	79.49	74.36	0.5385	0.7617	0.6517–0.8716	< 0.0001
Fluorescence imaging	–	82.05	69.23	0.5128	0.7564	0.6457–0.8671	< 0.0001
Pleural fluid cytological	–	100	30.77	0.3077	0.6538	0.5312–0.7765	0.0194

Pleural fluid cytological: traditional pleural effusion cytology examination and diagnosis; Fluorescence imaging: the fluorescence absorption of IR808@MnO in malignant cells; Fluorescence quantification: the fluorescence intensity of IR808@MnO

The data in this article demonstrate the low diagnostic efficacy of pleural fluid exfoliative cytology. This method relies entirely on the judgment of pathologists. Malignant cells exhibit changes in the nucleus and cytoplasm, including enlarged nuclei, nuclear deformities, hyperchromatism, and an imbalance in the nuclear-cytoplasmic ratio. The cytoplasm is relatively sparse and often contains phagocytosed foreign materials. Furthermore, malignant cells and mesothelial cells are morphologically very similar, making it particularly challenging to distinguish between them, especially when there are few detached cells in the pleural effusion. As shown in Fig. 7B and Table 2, the area under the ROC curve for pleural fluid cytology is 65.38%, with a specificity of 30.77, indicating a low diagnostic efficacy, which may be related to the capabilities of the hospital and the judgment skills of the pathologists.

Classical pleural fluid exfoliative cytology rarely interprets benign cells as malignant, but it is not as accurate as newer methods for interpreting malignant cells. However, the new method has an error in benign pleural effusions, and its large mean fluorescence values are mainly concentrated in patients with pyothorax, probably because the large amount of cell necrosis in pyothorax tends to cause fluorescence adsorption. The detection errors in living cells may be caused by unreliable variables including complicated intracellular interferences, uneven probe dispersion, and equipment setups [26]. Massive necrotic cell production in the pleural effusion of the pyothorax. This may be the cause of the very large deviations in values produced by pyothorax. Therefore, the new method has a good advantage in the diagnosis of malignant pleural fluid.

## Conclusions

Nowadays, pleural fluid exfoliative cytology, which is commonly used in clinical practice to qualitatively determine the benign and malignant nature of pleural fluid, varies in sensitivity and specificity due to the varying levels of pathology departments in different hospitals, but the overall value is on the low side. In this study, by cleverly embedding IR808 fluorescent dye in MnO nanoparticles, which increased the light stability and water solubility, and through the detection of near-infrared fluorescence in vivo with an in vivo imager, which greatly shortened the detection time from the taking of pleural fluid to the fluorescence validation, we only needed staining, fixation, and cleaning work for 1 h of time, which greatly reduced the time cost. Meanwhile, through statistical analysis, its sensitivity, specificity, positive predictive value, and negative predictive value, as well as the ROC curve, showed its superior diagnostic ability for malignant pleural fluid. Therefore, the IR808@MnO novel fluorescent probe is a time-saving, sensitive, and convenient clinical detection tool for malignant pleural fluid, which is worthy of clinical promotion.

## Data Availability

The processed data required to support the findings are included in the article. The datasets are available from the corresponding author on reasonable request.

## Abbreviations

CL1-5: Human lung adenocarcinoma cells
A549: Human non-small cell lung cancer
MDA-MB-468: Human breast cancer
U-87MG: Human glioblastoma
MKN-7: Human kidney cancer
Hela: Human cervical cancer
BEAS-2B: Human epithelial cells
HUVEC: Human umbilical vein endothelial cells
HSF: Human fibroblasts
VE: Human vascular endothelial cells
